# Efficient Evaluation of Wireless Real-Time Control Networks

**DOI:** 10.3390/s150204134

**Published:** 2015-02-11

**Authors:** Peter Horvath, Mark Yampolskiy, Xenofon Koutsoukos

**Affiliations:** 1 Department of Broadband Infocommunication and Electromagnetic Theory, Budapest University of Technology and Economics, Egry J. u. 18., Budapest H-1111, Hungary; 2 University of South Alabama, 150 Jaguar Drive, Mobile, AL 36688, USA; E-Mail: yampolskiy@southalabama.edu; 3 Institute for Software Integrated Systems, Vanderbilt University, 1025 16th Ave S, Nashville, TN 37212, USA; E-Mail: xenofon.koutsoukos@vanderbilt.edu

**Keywords:** cyber-physical systems, system simulation, wireless real-time networks

## Abstract

In this paper, we present a system simulation framework for the design and performance evaluation of complex wireless cyber-physical systems. We describe the simulator architecture and the specific developments that are required to simulate cyber-physical systems relying on multi-channel, multihop mesh networks. We introduce realistic and efficient physical layer models and a system simulation methodology, which provides statistically significant performance evaluation results with low computational complexity. The capabilities of the proposed framework are illustrated in the example of WirelessHART, a centralized, real-time, multi-hop mesh network designed for industrial control and monitor applications.

## 1. Introduction

Cyber-physical systems (CPS) represent the tight integration of physical systems with computing and networking capabilities. Networked control systems (NCS) are an important class of CPS, in which physical processes are controlled using networked sensors, controllers and actuators. Thus, the performance of these CPS directly depends on the predictability of the quality of the communication flows. Especially if such CPS are part of an industrial control and sensor application, the requirement of a very low packet drop rate under bounded communication delays is imposed on the networks.

As of today, the typical state-of-the-art real-time wireless networks are much more predictable than their more general, contention-based counterparts, owing to their static topology and, in general, pre-planned routing and scheduling mandated by the respective standards. A very well-established example of such an approach is WirelessHART [1], a widely-used wireless industrial control/sensor protocol that offers real-time guarantees for delay-sensitive applications. However, such wireless standards do not mandate specific algorithms for performance-critical tasks, such as scheduling of nodes and/or physical layer resources in the link layer or providing an end-to-end routing. In these cases, system designers are free to choose and implement their preferred algorithms and to take competitive advantage of their more sophisticated designs. Thus, it is indispensable for system designers to verify their proposed approaches over a broad range of scenarios with realistic network sizes in order to ensure the resilience and scalability of their algorithms and designs.

Verification of systems in general is a complex and involved procedure [2]. In our context, verification of the design choices can be done either by measurement on test beds or by simulations. The deployment of realistic testbeds is generally expensive. Moreover, even large testbeds cannot always accurately capture all of the possible channel conditions that a deployed system might encounter, e.g., fading over different channels. On the other hand, simulators are more flexible in this respect, but detailed packet-level simulators exhibit a high computational complexity, which results in limited scalability.

In this paper, we propose a novel simulation framework, which overcomes the above-mentioned limitations. This framework can simulate a centralized wireless real-time mesh network efficiently. Moreover, it provides a high accuracy network simulation and especially includes a realistic model of the physical layer with multi-channel frequency-hopping mesh networks. We have evaluated the proposed framework by implementing a system simulator for WirelessHART-based NCS.

The paper is organized as follows. In Section 2, we outline the challenges in the performance evaluation of wireless real-time mesh networks in the cyber-physical context. Section 3 describes the simulation framework and presents the general modeling techniques, especially with regard to the modeling of the performance of wireless links. Section 4 describes the implementation of the simulator and shows validation results. In Section 5, we present a specific system simulator design for WirelessHART-based industrial control networks to demonstrate the usefulness of our proposed approach.

## 2. Problem Description

**Figure 1.**
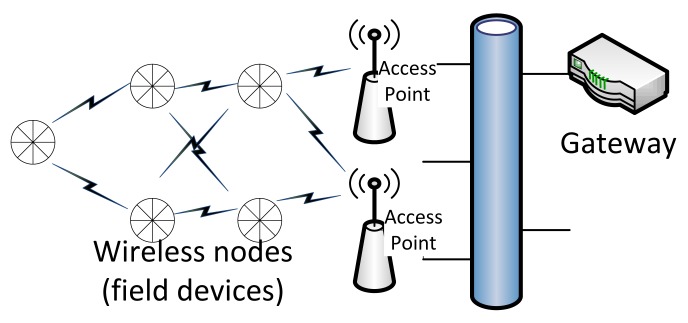
Common building blocks of an industrial mesh sensor/control network.

A typical centralized wireless real-time mesh network is illustrated in Figure 1. The network typically consists of a gateway node (GW), access points (APs) and field devices (FDs) performing sensor and actuator functionality. The GW provides bridging functionality to host applications and may also host the centralized network manager entity. The field devices connect to one or more access points, and applications (such as control algorithms) run on the gateway. A well-known example for such a centralized system is WirelessHART. In a typical, networked control application, the host application would process sensor measurements and provide control updates to the actuators.

### 2.1. Simulation Challenges

Having presented the main characteristics of the systems under consideration, in this section, we briefly summarize the major challenges in evaluating large-scale wireless real-time control networks.

#### 2.1.1. Accurate Models of the Environment

Signal fading is the primary manifestation of the effects of the wireless propagation channel. The fading manifests itself at multiple scales (large-scale fading due to path loss and shadowing effects and small-scale variations due to scattering effects). These fading manifestations might be correlated over time, space and frequency, ultimately determining the throughput and delay characteristics of the system. Furthermore, network simulation might assist system designers to assess the performance of the system under more diverse operating conditions for which real testbeds are available.

#### 2.1.2. Accurate Models of Link Performance

Error models mapped to the instantaneous signal-to-noise ratio (SNR) are often used in packet-level simulators, although this approach neglects some of the important effects caused by channel fading. Finite-state Markov models [3] are appealing from an analytic perspective, but they require separate parametrization for every possible combination of channel models and physical layer transmission schemes. Currently, they cannot cope well with frequency-selective fading channels, either. The most accurate results can be obtained by performing so-called Monte Carlo simulations of the actual transmitter and receiver transmissions, down to the level of modeling the transmission of individual bits and radio frames. Although computationally very intensive, this approach provides the highest fidelity, and the performance of specific receiver algorithms can be considered accurately, such as using coherent or noncoherent detection, *etc.* Finally, certain transmission schemes allow for computationally-efficient abstraction of the physical transmission [4,5]. This abstraction takes into account the instantaneous channel realizations, as well as the receiver characteristics, so that accurate error predictions can be obtained without performing bit-level simulations in the physical layer. Instead, one can statistically relate the probability for successfully transmitting entire frames to the instantaneous channel (fading) realization that characterizes the path between the transmitter and receiver. These approaches allow very good accuracy with comparatively low computational complexity, at the expense of some loss in the generality of the model [5].

Although current off-the-shelf wireless *ad hoc* and sensor technologies are based on low-complexity, non-adaptive PHY and MAC schemes (such as those based on the IEEE 802.15.4 standard), more recent developments have begun to find their way into low-complexity, low-cost sensor and industrial networks. Recent amendments to the IEEE 802.15.4 standard bring more sophisticated transmission techniques, for example coded, adaptive multi-carrier modulation in [6]. These techniques effectively mitigate or even exploit channel fading and open additional degrees of freedom (such as frequency-domain channel-dependent scheduling). However, efficient exploitation and realistic evaluation of such schemes call for more complex channel and link performance models, which conventional packet-level network simulators often do not incorporate.

#### 2.1.3. Simulation Efficiency/Scalability

Combining packet-level network simulators and simulators for the physical system is a convenient and less error-prone way of creating complex simulations for cyber-physical systems. This convenience, however, comes at the cost of scalability and simulation efficiency. Network simulations involving a large number of nodes are still challenging for detailed packet-level simulators. To gain comprehensive insight into the performance of the simulated system, potentially a large number of independent Monte Carlo trials need to be evaluated to obtain satisfactory confidence in the results for different network geometries, channel models and, specifically for cyber-physical systems, different physical disturbances that might affect the system. If there is a physical part to the simulated system, then the physical simulator requires appropriate integration and tight time synchronization with the network simulation engine. These requirements aggravate the complexity/scalability issues with detailed packet-level simulators. Therefore, for practical purposes, evaluation methods are needed that resort to abstraction mechanisms of acceptable complexity to decrease the simulation complexity, while not compromising the accuracy and credibility of the results. Furthermore, eliminating the synchronization overhead between coupled simulation engines might also lead to performance improvement in terms of simulator scalability.

### 2.2. Limitations of Existing Tools

In the literature, there appear different efforts and approaches addressing the performance evaluation of various wireless mesh networks. Specifically, we can find similar efforts for modeling WirelessHART. As an example, [7] extends the basic, general-purpose IEEE 802.15.4 model of the ns-2 (network simulator 2) simulator with a detailed WirelessHART stack, including accurate modeling of the network time synchronization and other network management tasks. The implementation inherits the transmission range-based physical layer performance model approach from the IEEE 802.15.4 model, *i.e.,* the success of a transmission only depends on the range between the corresponding nodes. Therefore, it does not aim at accurately modeling the lower layers. The ns-3 module in [8] adopts a simple two-state Gilbert–Elliot statistical model for packet errors without considering the underlying network. A different approach is taken by [9] by connecting a detailed packet-level network simulator (ns-2) and MATLAB Simulink for the dynamics simulations by using High Level Architecture (HLA) as the means of integration. Their efforts result in a more general, packet-level CPS evaluation framework. However, significant computational complexity results from the synchronization requirements between the two simulators, and accuracy is limited by the fairly simple physical layer models present in the ns-2 simulator.

## 3. Simulation Framework

Our proposed simulation framework for wireless real-time mesh networks exploits common design restrictions that render such networks significantly more predictable than contention-based wireless networks. The most important common design features of state-of-the-art real-time mesh networks include the following:

- Static, conflict-free resource scheduling: the physical layer resource usage is coordinated centrally, so that collisions are explicitly avoided, and thus, there is no interference (or only a very low amount) within nodes belonging to the same network. The spatial reuse of frequency resources is not allowed, and the nodes are assigned fixed transmission opportunities in time and frequency, depending on their pre-arranged transmission needs. Furthermore, limiting the maximum number of nodes within the network prevents problems arising from the lack of frequency reuse.
- Frequency division multiple access by coordinated frequency hopping: to increase the number of simultaneous transmissions, the nodes perform coordinated frequency hopping over multiple channels; thus, multiple nodes, up to the number of available channels, can transmit simultaneously. The frequency hopping also mitigates narrowband external interference and effectively randomizes the effects of the multipath fading in the wireless channel.
- Centrally-coordinated, redundant multipoint routing to avoid failures due to problems with single nodes within the network.

This static, mostly contention-free operation allows building simulators that are significantly less complex than typical simulators for wireless *ad hoc* networks and allows for significantly increased simulation accuracy by eliminating the intra-system interference issue. On the other hand, some design features of such real-time networks, such as channel hopping, are usually not present in other wireless systems.

System-level simulations focus on the network-wide aspects of complex wireless systems that are intractable by analytic means and/or do not lend themselves to define a single figure of merit to characterize the performance of the system. Such aspects in a typical wireless mesh, *ad hoc* or sensor network might include algorithms for user and resource scheduling, resource allocation, interference management, *etc.* In addition to these pure networking aspects, in wireless cyber-physical systems, the design and verification of the underlying physical system pose an important additional challenge.

Using realistic system simulators, the system-level effects of physical and link layer improvements can be evaluated without the need to implement the improvements on actual devices in the early design stages. Very detailed packet-level simulators, even if they incorporate accurate physical layer models, often yield to excessive simulation times to gain statistically significant results. For this reason, computationally efficient abstract evaluation techniques are preferred.

In this section, we present our system simulation framework for wireless real-time control networks, an approach that can yield accurate system-level application performance metrics, while keeping the computational complexity reasonably low by exploiting the inherent restrictions present in typical wireless real-time control networks.

### 3.1. Simulator Structure

**Figure 2.**
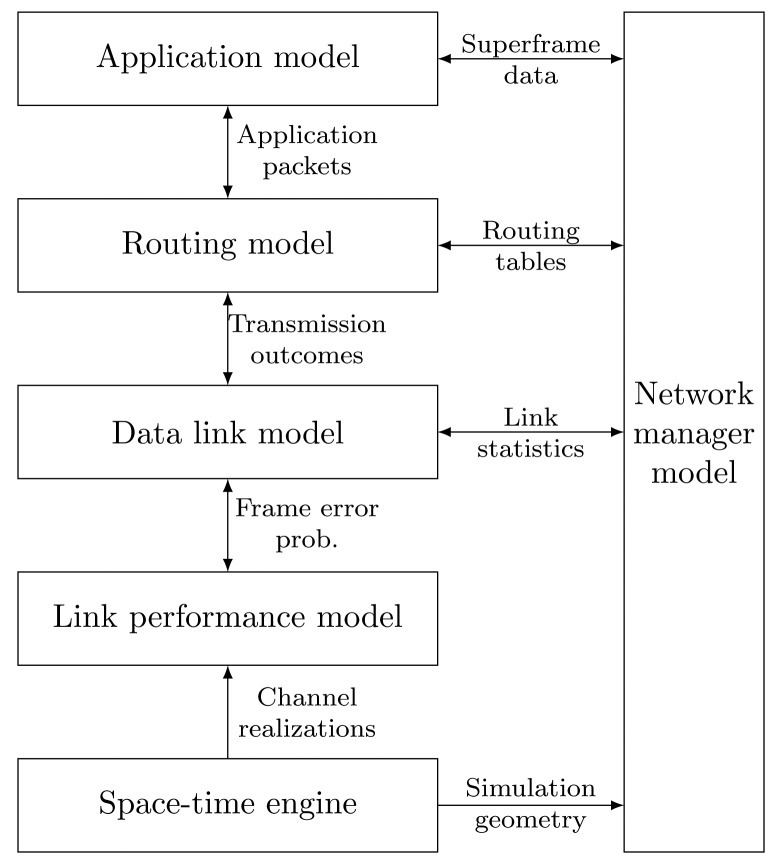
Main components of the system simulation framework.

Wireless mesh networks, *ad hoc* networks and sensor networks are rather diverse when it comes to their applications and the underlying networking technologies; therefore, devising a generally applicable system simulation model is more challenging. The layering in our framework roughly follows the conventional ISO/OSIlayer model, but, depending on the specific standard and the simulation goals, the partitioning of the functionalities within the layers does not necessarily follow the conventional layering. The main components of the proposed framework are shown in Figure 2. These are:

1. The application model, if present, models the behavior of the application associated with the system. Specifically, in the cyber-physical context, the application is often one or more networked control system consisting of plants, sensors, controllers and actuators.
2. The routing model specifies the end-to-end forwarding of packets within a multi-hop network.
3. Closely integrated with the routing model, the data link model is typically responsible for modeling the logical link control and the media access functionality within the respective wireless standard. In a wireless system, these functionalities include scheduling of the physical resources, queueing, link-level retransmission handling and providing multiple-access facilities. This model relies on the link performance model, which ultimately decides whether a certain link-level frame is successfully received by the respective receiver(s). In order to support systems relying on channel-dependent scheduling, this model can be complemented by the link measurement model that derives (either ideal or realistic) channel state information for the nodes, which typically will be used for scheduling users and/or transmission resources. In half-duplex sensor networks, these measurements are typically acquired from channel state feedback or by exploitingthe reciprocity of the wireless channel between two nodes.
4. The space-time engine provides the link performance model with instantaneous channel realizations, which are generated using the geometry of the scenario, according to the specified channel models.
5. Finally, the network manager module might contain functions pertaining to centralized network management or any kind of cross-layer information flow that relates the interoperability of the modules. It might also contain some functionalities required for managing the simulations.

We focus on proposing accurate, but efficient link performance models and realistic space-time models that can reproduce common propagation phenomena observed in wireless networks.

Our simulator has a lower layer focus, and it omits some details of the actual protocols, which conventional packet-level simulators model in full detail. Depending on the application, such details might consist of packet exchanges associated with network configuration, node synchronization, mobility, *etc.* Our simulator follows the “simulation drop” concept: the simulation consist of multiple, independent Monte Carlo drops. A drop means an independently-generated initialization set for the simulation parameters and the subsequent temporal evolution of the same simulation scenario, during which the scenario geometry and the large-scale fading parameters are kept constant. For a subsequent drop, the large-scale fading parameters and, depending on the simulation goals, the entire node deployment will be randomly re-generated [4]. The drop concept ensures that the results are representative and statistically significant for the investigated scenario and not only for a particular configuration. Omitting the detailed message exchange allows us to run a large number of drops with low computational complexity, despite the high fidelity of the link performance models and channel descriptions.

In most applications, we assume at the beginning of each simulation drop that the network has been configured.

### 3.2. The Space-Time Engine

Realistic system simulation involves generating wireless channel realizations according to a pre-defined, realistic channel model, which approximates the typical propagation conditions encountered in the scenarios of interest well. The channel fading is described by three components: large-scale fading consisting of a path loss due to distance-dependent average attenuation of the channel; a local random fluctuation around the mean signal level due to shadowing effects; and a small-scale fading due to the multipath and diffuse propagation effects [10].

The path loss over a wireless link is generated according to:

(1) $$L(dB)=L_{0}(d_{0})(dB)+10n\log(d/d_{0})+S(dB)$$

where *d*_0_ is a reference distance, *L*_0_(*d*_0_) is the average path loss at the reference distance, *d* is the link distance and *S* is a zero-mean Gaussian random variable with standard deviation *σ_S_*, expressed in dB [10]. The latter represents the effect of the shadowing on the link budget. The space-time engine allows us to take the spatial correlation of the shadow fading into account, *i.e.,* it enables the generation of non-independent shadow fading samples for geometrically closely-spaced wireless links according to some analytic or measurement model, such as exponential or the model described in [11].

There is an additional challenge in designing system simulation for a frequency-hopping mesh network that is not well addressed in the literature. Being such a system, WirelessHART employs a slot-by-slot frequency hopping scheme, *i.e.,* a transmission and an eventual retransmission between the same network nodes will take place on different RF channels. A realistic model needs to account for a varying degree of correlation between the fading processes experienced by a single link on different carrier frequencies at closely-spaced time instants. Intuitively, a large degree of frequency correlation (high channel coherence bandwidth) will render the frequency hopping ineffective at combating long, deep fades, whereas a low frequency correlation is beneficial, as successive transmission attempts experience nearly independent channel realizations, thus yielding to frequency diversity.

The existing literature does not provide reliable data on the frequency correlation in industrial environments. Measurement results for large indoor hotspots indicate that typical channels exhibit significant (>0.5…0.7) correlation over a bandwidth of 100 MHz for line-of-sight and 20 MHz for non-line-of-sight scenarios, respectively [12]. Thus, real channels might show significant correlation over multiple 5 MHz-wide 802.15.4 channels in an industrial indoor environment.

To address all of the mentioned challenges, it is necessary to adopt a low-complexity analytical multipath fading model that allows tight control of temporal fading correlation, specification of a certain channel delay profile and that provides small-scale fading realizations at multiple carrier frequencies, in which the frequency correlation is determined by the temporal support of the power delay profile. The basis of the model is the channel simulation model presented in [13]. The channel is described by a discrete tapped-delay line model:

(2) $$h_{i}(t,\tau )=\sum_{l=0}^{L-1}\mu_{l}^{\left(i\right)}(t)\delta (\tau -\tau_{l})$$

where *h_i_*(*t, τ*) is the time-variant channel impulse response on carrier frequency index *i, L* is the number of channel taps, $\mu_{l}^{\left(i\right)}$ is the time-variant tap coefficient that characterizes the fading on the carrier frequency *i* and *τ_l_* is the propagation delay of the *l*-th channel tap. It is reasonable to assume flat fading, as long as the root mean square (rms) delay spread is much less than the signaling interval (for example, Channel D would be flat fading for 802.15.4 g). Flat fading is the special case of Equation (2) with *L* = 1 tap, but still, the underlying power delay profile (PDP) [10] determines the fading correlation among multiple carrier frequencies.

In addition, we propose to modify the simulation model presented in [13] by changing the Doppler spectrum to Gaussian, which better approximates the behavior of measured indoor channels, which have been found to possess bell-shaped Doppler spectra [14] instead of following Jakes' model. This amounts to prescribing a temporal correlation for the fading taps according to:

(3) $$r_{\mu_{l}\mu_{l}}(\tau )=\frac{1}{\sqrt{2}}\exp\left(-\pi \frac{f_{c}}{\sqrt{\ln2}}\tau^{2}\right)$$

where *f_c_* is the −3 dB cutoff frequency. A practically realizable fading simulator results by setting the frequencies *f_i,n_* of (Equation (16a and 16b)) in [13] by solving the nonlinear set of equations:

(4) $$\begin{array}{cc} \frac{2n-1}{2N_{i}}-\text{erf}\left(\frac{f_{i,n}}{f_{c}\sqrt{\ln2}}\right)=0, & n=1,2,\ldots ,N_{i}-1 \end{array}$$

and:

$$f_{i,N_{i}}=\sqrt{\frac{\beta N_{i}}{{\left(2\pi \right)}^{2}}-\sum_{n=1}^{N_{i}-1}f_{i,n}}$$

where *β* = 2(π*f_c_*)^2^/ ln 2.

**Figure 3.**
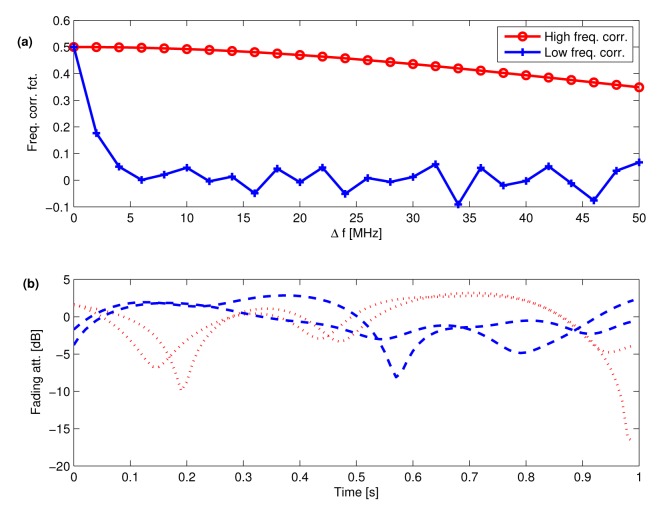
**(a)** Sample frequency correlation functions; (**b**) Sample pair of small-scale fading realizations at carriers spaced 50 MHz apart (dashed: High correlation; dotted: Low correlation).

Figure 3a shows the frequency cross-correlation functions of ℛ{*μ*_1_(*t*)} for pure Rayleigh fading (*K* = −∞ dB), whereas Figure 3b shows 2 fading realizations obtained 50 MHz (10 channels) apart for *L* = 1 for high and low frequency correlation, according to Figure 3a, assuming a 1.5-Hz Doppler spread.

### 3.3. Link Performance Model

In order to determine the packet loss probability for a given packet, one needs the mapping from the effective SNR to the packet error probability. In the simplest setting, these curves can be directly used to determine the packet error ratio if the fading in the channel can be regarded as approximately constant during the transmission of one block (quasi-static assumption), and the channel magnitude response is approximately constant over the transmission bandwidth (frequency-flat fading). If the latter assumption is not valid, the effects of the frequency selectivity need to be taken into account, which also depends on the signal processing algorithms employed in the respective receiver to cope with, or even exploit, the distinct multipath echos in the channel. This presents an additional non-trivial step in the performance evaluation for IEEE 802.15.4-like, relatively wideband, spread spectrum-based air interfaces. In cellular context, this challenge is usually tackled by applying various semi-empirical SNR penalties to the instantaneous SNR.

At this point, it should be noted that finding the packet loss probability, given an instantaneous channel realization and co-channel interference value, can be done in a more general way if the communication scheme employs coded orthogonal frequency division multiplexing (OFDM), as, for example, certain transmission modes of 802.11 do. OFDM, which uses low-rate parallel transmission on multiple subcarriers, “converts” the frequency-selective wireless channel into a set of parallel frequency-flat fading channels, and it is relatively simple to find an compression function that yields a scalar effective signal-to-noise ratio based on the per-subcarrier signal-to-noise and interference ratios (SINRs). If the compression function is chosen appropriately, the resulting effective signal-to-noise ratio marks a point on the AWGN packet error ratio curve (as in Figure 6 shown in Section 5), which approximates well the real packet error ratio that the multipath fading channels were exhibiting. Such mappings include exponential effective SINR mapping and mean instantaneous capacity mapping [4].

## 4. Simulator Implementation for WirelessHART Simulations

This section present a cyber-physical case study, namely we investigate the stability of a typical networked control system when the sensor measurements are transmitted over a WirelessHART-like wireless mesh network.

### 4.1. The WirelessHART System

WirelessHART represents the state-of-the-art in wireless industrial mesh networking [1]. Within the scope of the present paper, WirelessHART itself defines the lower four layers of the network stack. It provides end-to-end routing capabilities. The routing is determined by the network manager in the form of routing graphs. Routing graphs are propagated into field devices, and the packets carry a routing graph identification, so that intermediate devices can forward the packets to the appropriate next-hop node. The routing graphs are set up redundantly, so that every node is reachable over multiple routes, both in the upstream and downstream directions, if possible. This feature provides resilience against temporary single-link failures within the network. The construction of reliable routing graphs for WirelessHART is extensively discussed in [15].

The data link layer follows the principles of the time synchronized mesh protocol [16]. Communication is organized in a time division multiple access (TDMA) structure with a fixed 10-ms time slot length. The network maintains accurate slot timing and the network-wide absolute slot number (ASN).

WirelessHART relies on link layer acknowledgments. One complete transaction (*i.e.,* data transmission, acknowledgment and the necessary guard times, switching times, *etc.*) must fit into one timeslot. Network communication is organized into periodically repeating superframes. WirelessHART is statically scheduled in order to provide guarantees on the end-to-end delay in delay-sensitive applications. The deterministic network schedule is set up by the network manager, based on the communication requirements (superframes) and the statistics collected by the nodes. Only one node is allowed to transmit in a particular time slot on a single carrier frequency, which prevents collisions and the resulting uncertainties. Sub-schedules that define which node is eligible to transmit and which node(s) need(s) to wake up and listen within a particular time slot, are downloaded into the nodes. A salient feature, frequency hopping over at most 16 channels in the 2.4-GHz industrial, scientific and medical (ISM) band, each being 5 MHz wide, provides a very effective frequency diversity to randomize channel fading and mitigate external interference present on a channel. Additionally, channels experiencing significant interference might be blacklisted.

### 4.2. Physical Layer Technologies

Typical wireless real-time mesh networks, such as WirelessHART and ISA100.11a by the International Society of Automation (ISA), adopt the physical layer of the IEEE 802.15.4-2006 standard [17] almost unchanged, complementing it with a proprietary Medium Access Control (MAC) scheme that is better suited for time-critical applications than the dominantly contention-based 802.15.4-2006 MAC. In addition to the legacy IEEE 802.15.4-2006 physical layer (PHY), we also briefly describe one recent addition to this family of standards, one element of the IEEE 802.15.4 g-2012 standard [6]. These two PHYs together represent typical PHY designs that our simulator should be able to model adequately: the former is a single-carrier, spread spectrum scheme, whereas the latter is a multi-carrier one, using error correcting codes. We consider these two PHYs in the case studies.

**Figure 4.**
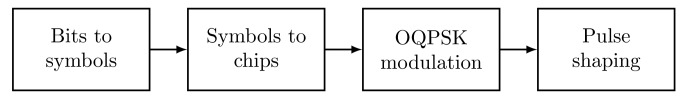
The IEEE 802.15.4 2.4-GHz PHY signal flow.

The standard IEEE 802.15.4 PHY for the 2.4 GHz band uses the offset quadrature phase shift keying (OQPSK)-based spread spectrum modulation technique. The transmit signal flow is illustrated in Figure 4. Blocks of four input bits are mapped to 32-chip sequences, which are, in turn, mapped to an offset-QPSK constellation. The subsequent pulse shaping uses half-sine pulses, so the resulting scheme can also be thought of as a constant envelope minimum shift keying (MSK) modulation. Typical receivers demodulate the signal either as MSK or by employing a coherent OQPSK demodulator.

The IEEE 802.15.4 g-based PHY: The 802.15.4 family of standards is being continuously amended with new PHYs for various specific purposes. Among these amendments, 802.15.4 g-2012 proposes three new PHYs primarily for wireless smart utility network purposes [6]. One of these new PHYs relies on multi-carrier transmission, orthogonal frequency division multiplex (OFDM). The techniques employed in this PHY make it a promising candidate for industrial environments, offering a significantly better spectrum efficiency than the original 802.15.4 PHY at comparable throughputs.

OFDM finds widespread usage in state-of-the-art wireless systems. Its well-known advantages include resiliency against frequency selective fading effects in the channel, low-complexity frequency domain equalization, good spectrum efficiency and fair tolerance against jamming/interference. Even more important for CPS applications where a minimum performance should be attained even under adverse channel conditions, it can be shown that the variance of the instantaneous link capacity decreases in a multipath fading channel as the number of subcarriers is increased [18]. This means that a well-designed OFDM-based system can ensure that an average channel capacity will be attained with high probability

The 802.15.4 g OFDM PHY mitigates typical channel impairments by the appropriate choice of modulation and channel coding schemes. OFDM with a cyclic prefix allows for low-complexity channel equalization, whereas convolutional coding contributes to the robustness of the PHY scheme. The PHY contains four “options” (parameter sets) that differ in the number of subcarriers and, hence, in the occupied bandwidth. These four options result in channel spacings from 200 kHz up to 1.2 MHz.

In the following, we focus on 802.15.4 g Option 2. This option uses 48 data subcarriers. The nominal channel bandwidth is 800 kHz, which is by a factor of six less than in the original 802.15.4, making this PHY significantly more efficient in terms of spectrum utilization. Additionally, IEEE 802.15.4 g allows adaptive modulation and coding with up to six different modulation/coding schemes. With Option 2, QPSK modulation with a rate 1/2 code, called Modulation and Coding Scheme 3 (MCS3), yields to a data rate of 400 kbit/s. An additional degree of diversity can be introduced by frequency repetition coding.

### 4.3. Simulator Implementation

**Figure 5.**
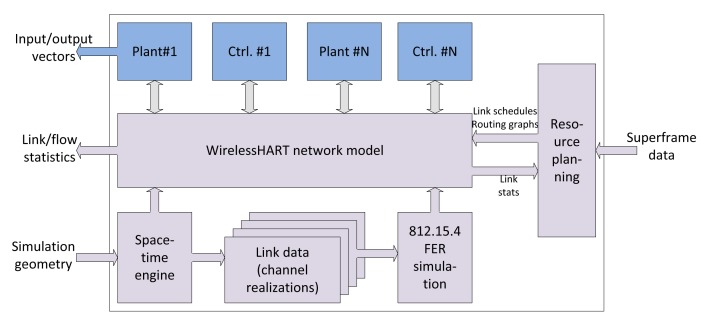
Functional blocks of the core WirelessHART simulator.

The implemented simulator is monolithic and written in the Python programming language. Its major functional blocks are illustrated in Figure 5. The simulator is governed by the SimPy discrete-event, process-based simulation engine. The process-oriented paradigm used by SimPy is slightly different from the more conventional event-oriented simulation approach primarily adopted in specialized network simulators (ns-2, OMNeT++). SimPy provides monitors for observing simulation events and interfaces well with NumPy/SciPy for numerical evaluation and the presentation of simulation results.

The user specifies the geometry and environmental parameters of the simulation, such as the locations of nodes or the parameters of the random distribution governing node locations, fading parameters, *etc.* Furthermore, the traffic parameters need to be specified, mainly the superframe data, *i.e.,* the period of the end-to-end transmission request between nodes, and routing graphs. The link schedules are obtained based on the superframe requirements and the routing graphs and packet loss statistics. Scheduling and routing planning operations are performed by external tools in an extensible manner. For example, a linear programming based tool determines the optimum routing based on the calculated per-link outage probabilities, and the link scheduling, in turn, is determined using this routing data. Alternatively, the routes could be set up based on empirically-collected link loss statistics after a short initial run of the drop.

Physical models in the system are also simulated in Python. The systems can be described either by state-space equations or by C language modules generated from Simulink models via the MATLAB Real-Time Workshop. The continuous-time state-space equations are numerically solved using the Assimulo Python package, which is, in turn, a wrapper around the C-based SUNDIALS (Suite of Nonlinear and Differential/Algebraic Equation Solvers) package. The Simulink-generated C source code contains the appropriate time-discrete solver. A straightforward Python wrapper is responsible for interfacing the Simulink-specific data structures and synchronization of the solver with the rest of the simulation. These wrappers are written in Cython.

The most computationally-intensive parts of the simulator are the wireless models, in particular the generation of channel realizations and the bit-level simulation to estimate whether the particular frame has been transmitted successfully according to the link performance model. These parts are implemented in C++ for efficiency and also wrapped using Cython to interface with the simulator core. These C++ parts are then called by the space-time engine and the WirelessHART model, respectively. The subsequently shown results have been obtained by simulating the transmitted packets by a bit-level simulator, and no link abstractions have been performed in order to obtain higher accuracy.

The simulator outputs discrete-time signal vectors (such as plant output signals and control signals) for debug and evaluation purposes as NumPy vectors. Furthermore, the WirelessHART network model keeps track of link-level and flow-level ratios of successful transmissions that can be queried after the completion of a simulation drop.

### 4.4. Simulator Validation

In this section, we show validation results to verify whether the link performance models accurately represent the real behavior of wireless devices. This also allows us to compare the legacy 802.15.4 and the 802.15.4 g OFDM PHY under realistic channel conditions. For this link-level evaluation, we use three multipath fading models: the frequency-flat assumption, 802.11n Channel Model D from [14] with 50 ns rms and a channel with a single exponential PDP, featuring a much higher rms delay spread than the 802.11n channels (so-called “Exp” channel, 1000-ns delay spread).

**Figure 6.**
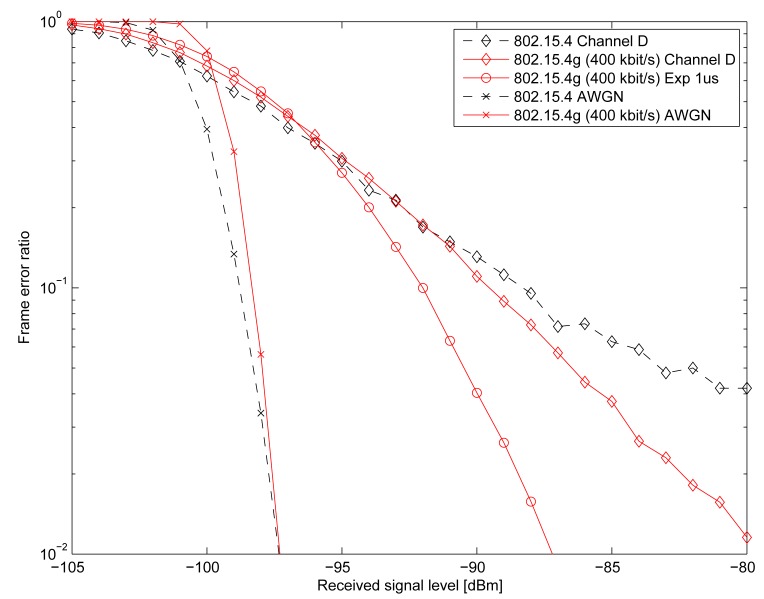
Frame error rate for 802.15.4 and 802.15.4 g Option 2 MCS3.

Figure 6 compares the link-level frame error rates for the legacy 802.15.4 PHY assuming a noncoherent correlation receiver, and the 802.15.4 g Option 2 MCS3 receiver, respectively. Transmit power is fixed at 0 dBm, and the size of the payload is 22 bytes. We can see that the non-fading (AWGN) performance is almost identical, whereas 802.15.4 performance starts to deteriorate even for the rather benign Channel D Rayleigh fading model. The 802.15.4 g receiver tolerates Channel D well and, as expected, can gain significant performance from the more frequency-selective “Exp” channel, benefiting from the fact that in the more frequency-selective channel, the subcarriers tend to fade more independently from each other. Therefore, the 802.15.4 g offers a higher data rate (400 kbit/s *vs.* 250 kbit/s) at 1/6th transmission bandwidth and significantly better performance in dispersive channels.

## 5. Case Study: NCS Performance over WirelessHART

In this section, we demonstrate the abilities of the system simulator described in the previous section by adopting a typical CPS benchmark, a networked industrial feedback control system. We study the application performance both with the standard physical layer and by considering a more spectrum-efficient alternative physical layer technology.

### 5.1. Control System Model

Although, often, fast dynamic systems are used for NCS performance evaluations, such as inverted pendulums [19], state-of-the-art wireless mesh networks are unable to to provide an adequately low latency for such applications. We use a classical example from the network control system literature, a two-input, two-output open-loop unstable batch reactor model [20], which is still highly delay-sensitive, but is feasible for control using sensor data obtained from wirelessly connected sensors. The plant is characterized by the following linearized state-space model:

(5) $$\text{ẋ}=\left[\begin{array}{cccc} 1.38 & 0.2077 & 6.715 & 5.676\\ 0.5814 & 4.29 & 0 & 0.675\\ 1.067 & 4.273 & 6.654 & 5.893\\ 0.048 & 4.273 & 1.343 & -2.104 \end{array}\right]\text{x}+\left[\begin{array}{cc} 0 & 0\\ 5.679 & 0\\ 1.136 & -3.146\\ 1.136 & 0 \end{array}\right]\text{u}$$

and:

(6) $$\text{y}=\left[\begin{array}{cccc} 1 & 0 & 1 & -1\\ 0 & 1 & 0 & 0 \end{array}\right]\text{x}$$

where ***x***, ***y*** and ***u*** denote the state, input and output vectors, respectively.

**Figure 7.**
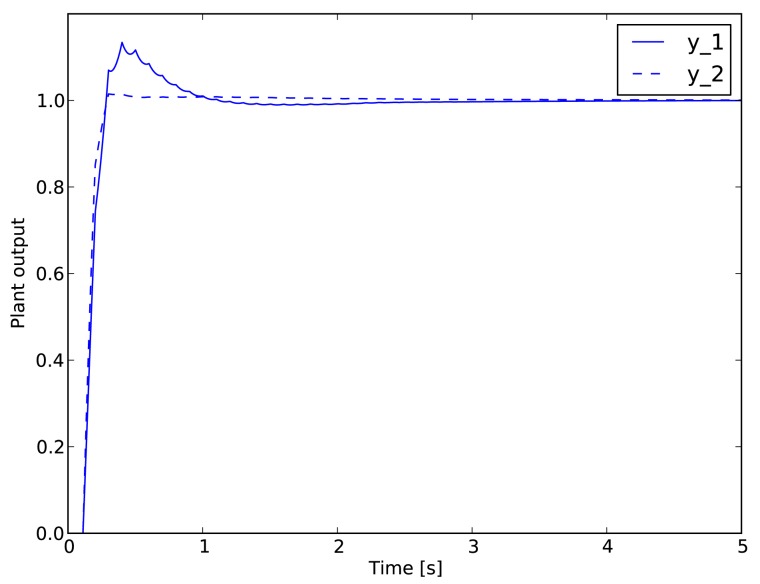
Step response of the batch reactor with the model predictive controller (MPC) controller.

Although traditional standard benchmarks, such as the one in [20], assume that the plant is controlled by a fixed proportional-integral (PI) controller, in this paper, we use a model predictive controller (MPC). This kind of controller, with its variable time horizon, is generally more robust and provides better control performance with increased tolerance against the delays and outages of the wireless transmission; therefore, it is more relevant for today's practical deployments than the static PI controller often used for this particular benchmark. As in [20], we assume that only sensor measurements are transmitted over the wireless networks, whereas the controller output is connected to the actuators using a wired connection having negligible delay compared to the delays occurring in the wireless network. The step response of the controlled system with negligible delays in both directions is illustrated in Figure 7. The controller is designed with the following parameters: the control interval is 100 ms; the prediction horizon is 500 ms; and the actuation horizon is 200 ms.

### 5.2. Stability of the Benchmark NCS

**Figure 8.**
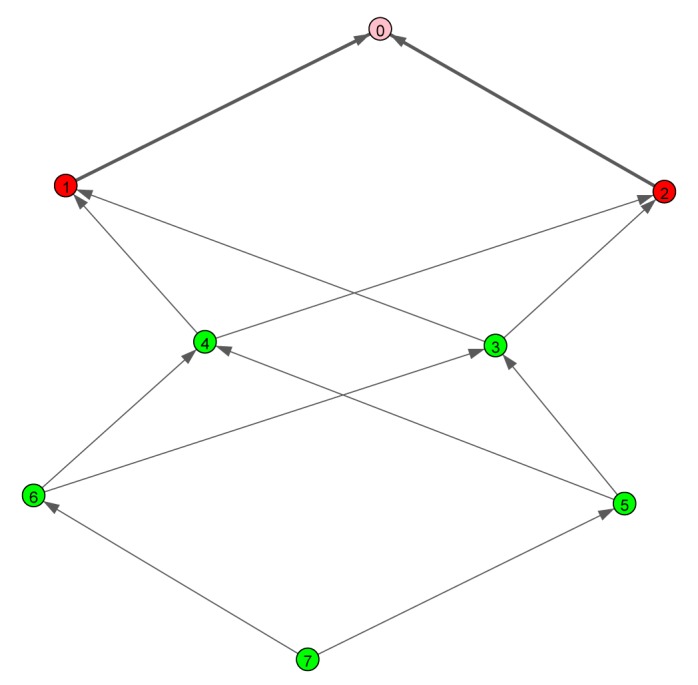
Example uplink graph. Node 0 is the gateway node (GW); Nodes 1–2 are access points (APs); Nodes 3–7 are field devices. Node 7 is the sensor.

Firstly, we investigate the effects of different channel models on the stability of the batch reactor setup. The uplink graph is shown in Figure 8, with Node 7 being the sensor node; thus, the gateway is reachable within three wireless hops. The simulation parameters are summarized in Table 1. We compare the successful packet delivery ratios and the ratio of the stable outcomes for the batch reactor, with three different Ricean *K*-factors and three different frequency correlation values (the independent block fading channel, the frequency-correlated channel with low correlation, *σ_τ_* = 2 ns, and the frequency-correlated channel with high correlation, *σ_τ_* = 100 ns.) We run every 10 s-long independent simulation trial 500 times. In order to assess the simulation efficiency, each run takes approximately one second on an Intel Xeon CPU running at 3 GHz.

To assess the NCS performance, we show two figures of merit for the experiments. The probability of successful delivery is the ratio of packets that arrive to the intended recipient (plant or controller) within the deadline that we specify in advance. This is a traditional, purely network-centric quantity. The ratio of the stable outcomes shows the probability for the NCS to remain stable within the investigated time horizon. The two quantities are clearly related, as a high delivery probability within a well-chosen deadline would result in a well-behaved control loop.

**Figure 9.**
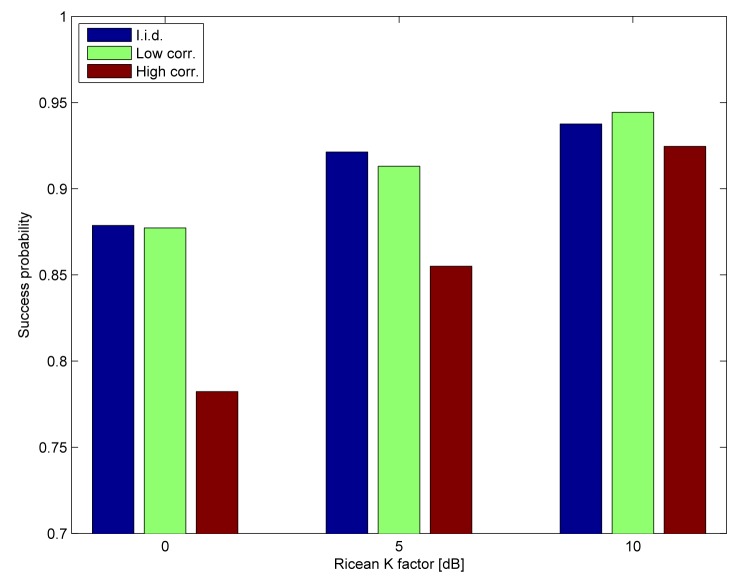
Successful delivery ratio for the configuration according to Table 1.

**Figure 10.**
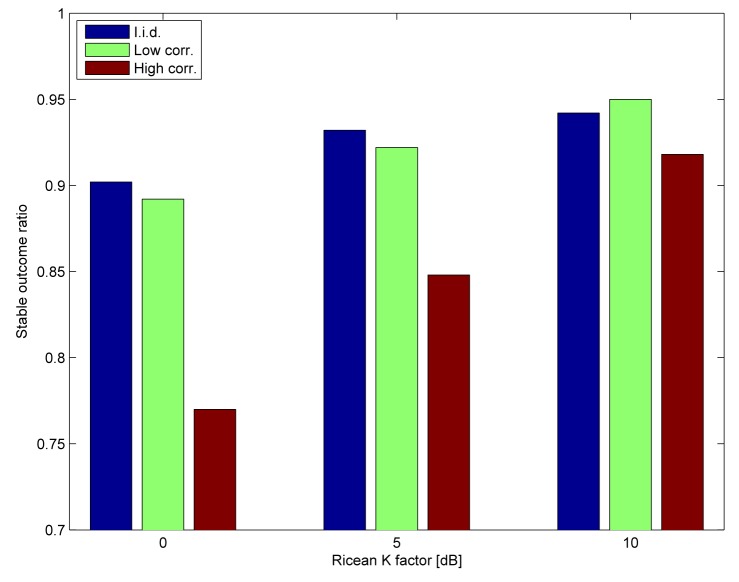
Ratio of stable outcomes for the configuration according to Table 1.

We consider a non-redundant routing scenario, *i.e.,* only Nodes 7, 5, 3 and 2 are activated. The deadline is set to six time slots (60 ms), such that every link can be activated twice (e.g., 7 → 5, 5 → 3, 3 → 2, 7 → 5, 5 → 3, 3 → 2). The successful delivery ratios are shown in Figure 9. As expected, the low correlation channel and the i.i.d. channel perform similarly, whereas the delivery ratios are adversely impacted in the highly correlated channel, with diminishing impact as the Ricean *K* factor increases. Figure 10 shows the ratio of stable outcomes under the same assumptions. The stable outcome ratio closely follows the successful delivery ratios.

**Figure 11.**
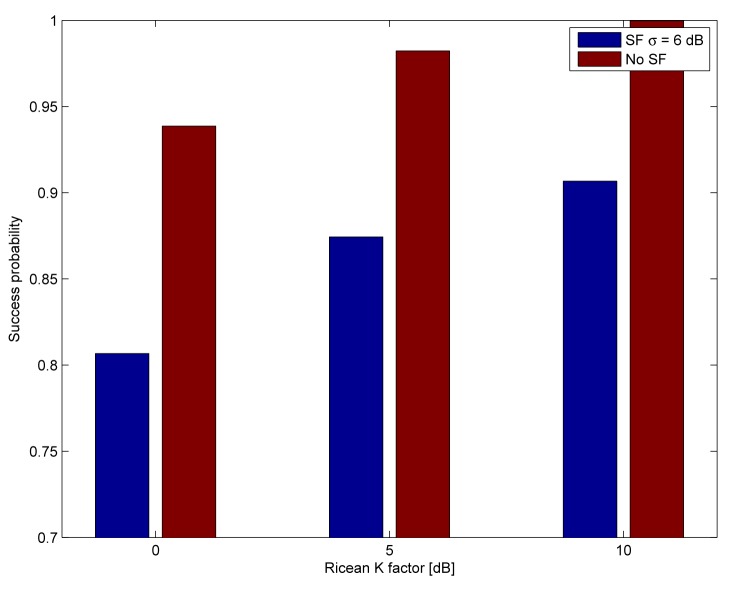
Successful delivery ratio with and without shadowing for the highly frequency-correlated channel.

**Figure 12.**
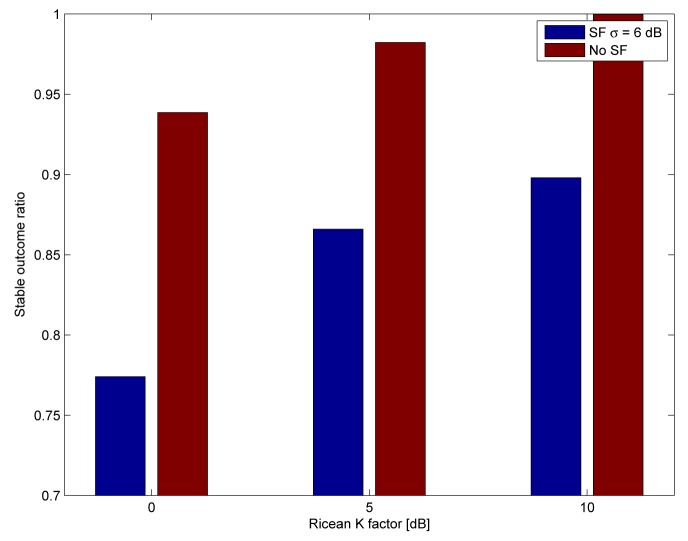
Ratio of stable outcomes with and without shadowing for the highly frequency-correlated channel.

Finally, we compare the effects of the shadow fading on the success rates and the stable outcomes by disabling the shadow fading compared to the parameters in Table 1. We only show the outcomes for the highly frequency-correlated channel. The success rates and the ratio of stable outcomes are shown in Figures 11 and 12, respectively. Increasing the standard deviation of the shadow fading deteriorates the performance considerably, although it yields to the same improvement or the decrease of the link budget with the same probability High frequency correlation and high shadow fading correlation together cause the largest degradation in performance.

### 5.3. Study of Alternative Physical Layer Technologies

**Figure 13.**
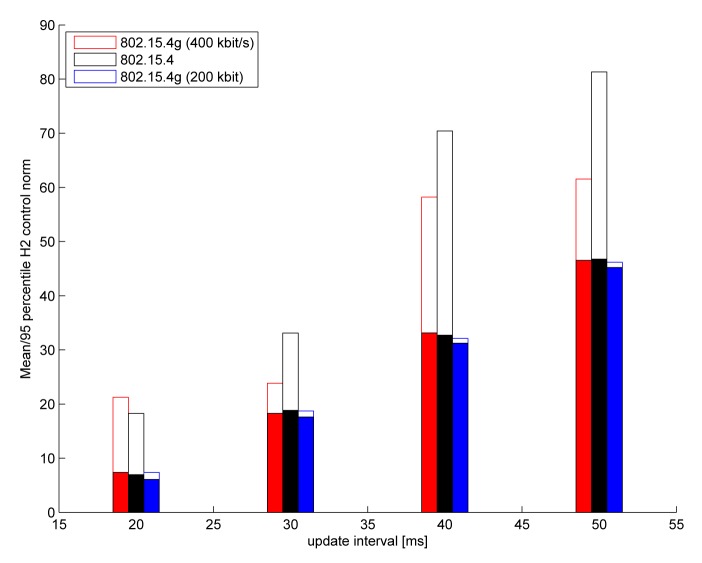
Quadratic control error *vs.* the update period (solid bars: mean error over the stable outcomes; empty bars: 95th percentile error).

In this part, we demonstrate the ability of the simulation framework to compare different physical layer options. In particular, we run the batch reactor benchmark over both legacy 802.15.4 PHY and over two different 802.15.4 g-based PHYs (both the 200-kbit/s and 400-kbit/s modes) and compare the ratio of stable outcomes between the two simulations. All of the simulation parameters are identical to those in Table 1; only the K-factor is increased to 10 dB, making the channel fading less pronounced. The quadratic control error (with respect to the impulse response of the delay-free feedback loop with a 10-ms sample interval) is shown in Figure 13 for the high-correlation flat fading channel, for sample intervals between 20 ms and 50 ms. Unstable outcomes are excluded from the mean error calculation, but we provide the 95th percentile error over all 100 independent drops. The 802.15.4 g 200 kbit/s PHY outperforms the other two PHYs; the feedback system remained always stable, and there is very little variation in the control error over the runs.

## 6. Conclusions

In this paper, we propose the application of the system simulation approach to accurately evaluate the performance of centralized real-time wireless mesh networks. Our framework considers accurate models of the wireless propagation and the peculiarities of the physical layer transmission schemes and receiver algorithms. We show that integrating models of the physical part (plants, controllers) is straightforward, and the integrated simulation simplifies simulation problems between the respective domains. Our case studies confirm the importance of realistic wireless models to assess the performance of wireless networked control systems.

## Figures and Tables

**Table 1.** Simulation assumptions for the batch rector trials.

**Parameter**	**Value**
Reference distance *d*_0_	15 m
Reference path loss *L*_0_(*d*_0_)	65 dB
Path loss exponent *n*	2.5
Ricean *K* factor	{0, 5, 10} dB
Shadow fading *σ_S_*	6 dB
Inter-node distance	130 m
Sample size	166 bits
Number of drops	500
